# Geospatial inequalities in severe leprosy disability in Pernambuco before and during COVID-19

**DOI:** 10.1016/j.bjid.2026.105968

**Published:** 2026-10-01

**Authors:** Andrezza Tayonara Lins Melo, Pauliana Valéria Machado Galvão, Carolina da Cunha Correia

**Affiliations:** Universidade de Pernambuco (UPE), Programa de Pós-Graduação em Ciências da Saúde, Recife, PE, Brazil

**Keywords:** Health indicators, Leprosy, COVID-19, Northeast Brazil, Ecological study

## Abstract

**Objective:**

The present study therefore aimed to compare and contextualize leprosy indicators in the State of Pernambuco during the pre-pandemic and pandemic periods, to assess the burden of disease using Years Lived with Disability, and to examine its distribution across Pernambuco’s various health regions.

**Methods:**

This ecological study used secondary data to compare epidemiological indicators and the burden of leprosy-related disease in the State of Pernambuco, Brazil, before (2017‒2019) and during (2020‒2022) the COVID-19 pandemic, and to examine its distribution among health regions.

**Results:**

The detection rate of new cases decreased from 33.44 to 24.54 per 100,000 inhabitants, although endemicity remained high in most regions and hyperendemic in Petrolina. The detection rate of new cases with grade 2 disability increased from 7.22 to 8.64 per 1000,000 inhabitants, particularly in the regions of Recife, Caruaru, and Petrolina. Years lived with grade 2 disability increased from 352.76 to 463.06 years.

**Conclusion:**

During the period from 2020 to 2022, the COVID-19 pandemic was associated with significant changes in most leprosy indicators, suggesting an increase in morbidity in the state of Pernambuco, Brazil. Despite the reduction in new leprosy cases due to mobility restrictions, the overburdening of the public health system, and the suspension of active case finding, particularly affecting children and vulnerable populations, there was an increase in hidden prevalence, treatment delays, and disabilities. The behavior of the epidemic in the state of Pernambuco was similar to that observed in Brazil, reinforcing the country's position as the second most affected by leprosy in the world.

## Introduction

Leprosy remains a significant public health challenge and a leading cause of preventable physical disability worldwide. Despite decades of control efforts, the disease persists in endemic pockets, particularly in tropical and subtropical regions. The Physical Disability Grade (PDG), classified from 0 (no impairment) to 2 (visible impairment), reflects sensorimotor impairments affecting the eyes, hands, and feet, and serves as a critical indicator of diagnostic delay and program performance.^1^^,^^2^

The endemic distribution of leprosy, together with its potential for functional and aesthetic complications, underscores the importance of disease control and epidemiological surveillance.^3^^,^^4^ Evaluation of leprosy indicators enables us to determine the epidemiological situation in any given region, thereby facilitating the development of targeted public policies, expanding the access to medical treatment, and improving the quality of care.^5^

Sustainable Development Goal-3 (SDG-3) of the United Nations (UN) 2030 Agenda aims to ensure healthy lives and promote well-being for all at all ages. Target 3.3 specifically calls for the elimination of neglected tropical diseases such as leprosy.^6^ Brazil’s national strategy to control leprosy incorporates various indicators, including the New Case Detection Rate (NCDR) and the NCDR of grade 2 Disability (G2D), enabling monitoring of progress toward elimination targets.^7^ Leprosy is also included in the Global Burden of Disease (GBD) study, which provides comprehensive estimates of the loss of health attributable to diseases and injuries across all countries and age groups.^8^^,^ ^9^

The impact of illness can be measured using Years Lived with Disability (YLDs), both globally and regionally.^10^ YLDs quantify disease-related disability and provide a comprehensive assessment of the nonfatal consequences of the disease.^10^^,^^11^ Despite Brazil’s position as the country with the second highest absolute number of new leprosy cases globally, the application of YLD methodology to leprosy remains limited, particularly in the context of the COVID-19 pandemic, which significantly disrupted public health systems.^12^, ^13^, ^14^

During the pandemic, Brazil has shown an unequivocal and dramatic decline in the number of new cases, attributable to mobility restrictions, the overburdening of the healthcare system and suspension of active case-finding activities. The national annual detection rate fell from 14.52 to 7.59 cases per 100,000 inhabitants (47.7%).^15^ However, reduced diagnoses do not reflect a true decrease in disease burden; rather, represents a diagnostic paradox- fewer detected cases but more severe disease at diagnosis.

The state of Pernambuco presented the highest number of leprosy cases in the Northeast Region in 2019 and 2021,^16^^,^^17^ with the 7th-highest detection rate in children under 15 and the 8th-highest overall detection rate in the country.^18^ The present study therefore aimed to compare and contextualize leprosy indicators in the State of Pernambuco during the pre-pandemic (2017‒2019) and pandemic (2020‒2022) periods, to assess the burden of disease using YLDs, and to examine its spatial distribution across Pernambuco’s various health regions.

## Methodology

We conducted an ecological study using data from Brazil’s Notifiable Diseases Information System (SINAN) for all leprosy cases reported in Pernambuco State. The study period was divided into pre-pandemic (2017‒2019) and pandemic (2020‒2022) phases.

Annual detection rates per 100,000 population and grade 2 disability rates per 1000,000 population were calculated for each period and health region. Population denominators were obtained from IBGE intercensal estimates.

Together with population data on state residents, by health region, case data were used to calculate specific leprosy health indicators.

The indicators were calculated following the definitions of purpose recommended by the Ministry of Health:

a) New Case Detection Rate (NCDR) per 100,000 inhabitants: was calculated to assess the magnitude and temporal trend of leprosy morbidity. It was computed by dividing the number of newly detected cases among residents of a given location during the year of evaluation by the total population of that location during the same period, then multiplying the result by 100,000. A rate lower than 2/100,000 is considered low, 2‒9.9/100,000 medium, 10‒19.99/100,000 high, 20‒39.99/100,000 very high, and higher than 40/100,000 hyperendemic.
b) New case detection rate with G2D (NCDR G2D) per 100,000 inhabitants: was calculated to evaluate the burden of disability caused by leprosy in the general population. It was computed by dividing the number of new cases with G2D residing in a given location and diagnosed during the year of evaluation by the population residing in the same location during the same period, and multiplying the result by 1000,000. There are no universally established reference thresholds for this indicator.
c) Years Lived with Disability (YLD): is a key component of Disability-Adjusted Life Years (DALYs), where DALY=YLL+YLD; YLD=I×DW×L, where I: Number of incident (new) cases in the population; DW: Disability weight (0 = Perfect health; 1 = Equivalent to death); L: Average duration of the case until remission or death (in years). YLDs were calculated using the disability weight assigned to the condition, which reflects its severity on a scale from 0 to 1. The disability weights applied were 0.011 for Grade 1 Disability (G1D) and 0.067 for G2D.^19^

### Data sources and mensuration

Secondary data were obtained from the publicly accessible and anonymized Notifiable Diseases Information System (SINAN).

### Statistical analysis

A descriptive analysis of sociodemographic variables and epidemiological indicators was conducted using absolute and relative frequencies, as well as measures of central tendency.^20^ Categorization and calculation of the indicators were performed using R statistical software (version 3.4.1), in accordance with the recommendations of the Brazilian Ministry of Health. Associations between categorical variables were assessed using Pearson’s chi-square test or Fisher’s exact test, as appropriate. For trend analysis, the Annualized Percentage Change (APC) was calculated based on two time points (the beginning and the end of the period), a method that requires fewer assumptions and is less susceptible to bias in larger populations. For YLDs, normality was assessed using the Shapiro-Wilk test. As the data did not exhibit a normal distribution, the Wilcoxon test was applied for comparisons between independent samples.^21^

### Ethical considerations

The study was approved by the Research Ethics Committee of the HUOC/PROCAPE Hospital Complex, under protocol numbers 6837,316 and CAAE: 79,410,524.9.0000.5192.

## Results

The present study analyzed all cases of leprosy reported in the State of Pernambuco between 2017 and 2022, including 9529 (57.90%) new cases reported from 2017 to 2019, and 6929 (42.10%) reported between 2020 and 2022 (Table 1).

**Table 1:** Sociodemographic and clinical characteristics of newly reported leprosy cases in the State of Pernambuco, 2017‒2019 and 2020‒2022.

Variables	Pre-pandemic		Pandemic		*p*-value
	2017‒2019		2020‒2022		
**Gender**	**n**	**%**	**n**	**%**	
Male	4978	52.24	3645	52.60	**0.6439**^a^
Female	4551	47.75	3284	47.39	
**Age**	**n**	**%**	**n**	**%**	**<0.0001**^a^
0‒15 years	605	6.34	313	4.51	
40‒60 years	3704	38.87	2800	40.41	
> 60 years	2173	22.80	1807	26.07	
**Race/skin-color**	**n**	**%**	**n**	**%**	
White	1778	18.65	1317	19.00	**0.5799**^a^
Black	1318	13.83	951	13.72	
Asian	74	0.77	45	0.64	
Mixed Race	5455	57.24	4002	57.75	
Indigenous	44	0.46	36	0.52	
Not known	860	9.02	578	8.34	
**Education**	**n**	**%**	**n**	**%**	
Not known	3206	33.64	2738	39.51	**<0.0001**^a^
Illiterate	726	7.61	408	5.88	
Elementary education	3608	37.86	2227	32.14	
High school	1575	16.52	1152	16.62	
Higher education	414	4.34	404	5.83	
**Degree of disability**	**n**	**%**	**n**	**%**	
Grade 0	5072	53.23	3239	46.75	**<0.0001**^a^
Grade 1	1756	18.43	1580	22.80	
Grade 2	689	7.23	600	8.66	
Not evaluated	2012	21.11	1510	21.79	
**Clinical form**	**n**	**%**	**n**	**%**	
Paucibacillary	2609	27.38	1357	19.58	**<0.0001**^b^
Multibacillary	6919	72.61	5571	80.40	
Not known	1	0.01	1	0.01	
Total	**9529**	**57.89**	**6929**	**42.10**	

a Pearson's Chi-Square test with Yates’ continuity correction.

b Fisher's exact test.

The NCDR in the State of Pernambuco continued to be classified as very high in both periods analyzed (33.4 per 100,000 population in 2017‒2019 and 24.5 per 100,000 in 2020‒2022), as shown in Table 2.

**Table 2:** NCDR of leprosy in the State of Pernambuco, 2017‒2019 and 2020‒2022.

Health regions	2017‒2019		2020‒2022		APC^a^	*p-*value^b^
	Rate	Classification	Rate	Classification		
Recife	44.05	Hyperendemic	30.34	Very high	−17.85%	<0.001
Limoeiro	24.17	Very high	20.23	Very high	−53.33%	0.02
Palmares	17.92	High	11.78	High	−42.23%	<0.001
Caruaru	12.63	High	10.58	High	−9.26%	0.01
Garanhuns	11.11	High	7.89	Medium	−27.8%	0.003
Arcoverde	17.65	High	10.41	High	−53.58%	<0.001
Salgueiro	28.32	Very high	18.57	High	−19.58%	0.004
Petrolina	76.2	Hyperendemic	67.2	Hyperendemic	7.53%	0.005
Ouricuri	42.04	Hyperendemic	31.87	Very high	−12.37%	<0.001
Afogados da Ingazeira	17.77	High	9.34	Medium	−57.01%	<0.001
Serra Talhada	30.14	Very high	23.21	Very high	−1.24%	0.01
Goiana	26.71	Very high	21.06	Very high	−38.57%	0.01
Total	33.44	Very high	24.5	Very high	−17.91%	<0.001

a Annualized percentage change: calculated from the ratio of the rate in 2022 to that in 2017, following Fay et al. (2006).

b Pearson's Chi-Square test with Yates’ continuity correction.

The average NCDR of G2D was 7.22 per 1 million population in 2017‒2019 and 8.61 per 1 million population in 2020‒2022. The results are presented in Table 3.

**Table 3:** G2D new case detection rate by health region, Pernambuco, 2017‒2019 and 2020‒2022.

Health regions	2017‒2019	2020‒2022	APC^a^	*p*-value
Recife	7.15	8.55	37.88%	0.008^b^
Limoeiro	8.68	5.47	−86.89%	0.019^b^
Palmares	4.87	3.62	−82.72%	0.192^b^
Caruaru	6.55	11.91	65.19%	0.055^b^
Garanhuns	6.23	9.09	−83.24%	0.999^c^
Arcoverde	4.39	4.23	−39.68%	0.442^b^
Salgueiro	8.61	7.28	9.44%	0.481^b^
Petrolina	7.75	11.44	71.25%	0.049^b^
Ouricuri	7.56	8.49	107.97%	0.308^c^
Afogados da Ingazeira	10.23	8.51	2.08%	0.305^b^
Serra Talhada	12.24	3.63	−79.95%	0.001^b^
Goiana	4.75	5.31	24.54%	0.849^b^
Total	7.22	8.61	21.53%	<0.001^b^

a Calculated from the ratio of the rate in 2022 to that in 2017, following Fay et al. (2006).

b Pearson's Chi-Square test with Yates' continuity correction.

c Fisher's exact test.

The analysis of total YLDs showed that leprosy accounted for 352 YLDs in Pernambuco during the pre-pandemic period. During the pandemic (2020‒2022), this figure increased to 463.06-years, primarily due to G2D cases. The YLD burden attributed to G1D was lower. However, when the rates were standardized per 100,000 inhabitants, a decrease was observed between the two periods, except in the Petrolina region (Table 4).

**Table 4:** Distribution of YLDs per 100,000 population by health region, State of Pernambuco, 2017‒2019 and 2020‒2022.

Health Regions	YLD1		p-value	YLD2		*p*-value
	2017‒2019	2020‒2022		2017‒2019	2020‒2022	
Recife	24.29	12.48		282.45	251.58	
Limoeiro	1.58	1.48		18.69	5.94	
Palmares	0.84	0.26		2.36	3.32	
Caruaru	1.43	0.66		3.6	21.97	
Garanhuns	0.13	0.15		1.4	3.13	
Arcoverde	0.32	0.03		0.85	0.78	
Salgueiro	1.1	0.27	0.027^a^	5.31	4.34	0.588^a^
Petrolina	8.00	26.28		122.81	486.81	
Ouricuri	2.8	1.42		26.38	33.32	
Afogados da Ingazeira	0.39	0.11		4.51	1.81	
Serra Talhada	2.14	0.38		19.02	3.4	
Goiana	0.94	0.64		4.54	7.28	
Total	36.77	23.98		352.56	463.06	

a Wilcoxon rank-sum test.

## Discussion

The present study shows that although the number of cases decreased during the initial years of the pandemic, the leprosy incidence remained high throughout both periods evaluated. The largest proportion of cases in both periods occurred among men aged 40‒60 years, individuals of mixed race, and those who had not completed secondary education. Internationally, the epidemiological profile of the disease, as observed in various countries, including India and China, indicates that most cases occur among women and frequently involve the Multibacillary (MB) form of the disease.^22^

A similar finding was reported in the study by Paz et al.,^23^ which observed an increase in multibacillary cases diagnosed between 2015 and 2020. A higher number of multibacillary cases is associated with a longer disease course, contributing to an increased occurrence of physical disabilities due to greater neural involvement and delays in diagnosis and treatment.^23^ In Brazil, leprosy presents a heterogeneous distribution across states, including those in the North and Northeast Regions,^24^^,^^25^ a pattern likely related to social vulnerability and spatially overlapping factors.^23^

Leprosy continues to disproportionately affect populations exposed to adverse socioeconomic determinants. Social vulnerability may likewise contribute to the high endemicity observed in the State of Pernambuco, with >40% of the population living below the poverty line.^26^ In the present study, although an overall reduction in the new case rate was observed during the pandemic, the Petrolina region remained hyperendemic in both periods. Endemicity decreased across the State by 17.91%, with Petrolina being the only municipality to present an increase (7.53%).

The persistence of hyperendemicity in Petrolina may be related to the municipality’s historically high burden of leprosy and the concentration of high-risk areas associated with social vulnerability. Previous studies have identified high to hyperendemic areas in the urban territory of Petrolina, suggesting persistent transmission and the need for strengthened surveillance and early case detection. These pre-existing epidemiological conditions may have contributed to the municipality maintaining high endemicity even during the COVID-19 pandemic, when changes in health service access and disease surveillance affected leprosy case detection.^27^^,^^28^

Despite the decline in reported cases during the early years of the COVID-19 pandemic, leprosy remained at a very high endemicity level in Pernambuco.^24^ This reduction in case detection may be associated with the pandemic, which placed considerable strain on health systems and services, thereby affecting disease surveillance and control.^24^ Notably, the NCDR of G2D increased significantly during the pandemic period (21.53%), suggesting delays in diagnosis and more serious sequelae. Surprisingly, significant increases in this indicator in regions such as Recife, Caruaru and Petrolina (*p* = 0.008, 0.055, and 0.049, respectively), reaching levels comparable to the Brazilian national average reported for 2019.

The present study is among the first to assess YLDs in Pernambuco in the specific context of the COVID-19 pandemic. The burden of leprosy was greater among cases with G2D, with an increase observed during the early years of the pandemic, particularly in the Recife and Petrolina regions, which recorded the highest absolute values. In contrast, the YLDs attributable to G1D decreased during the same period. Although G1D reflects sensory impairment with consequences for quality of life, these manifestations may be less likely to generate increased demand for health services. The elevated YLD levels observed in the Petrolina region may be influenced by its proximity to highly endemic areas and by intensified surveillance efforts, which may increase case detection.^29^^,^^30^

National data help to contextualize these findings. One Brazilian study reported 1213 YLDs in 1990, and 2515 YLDs in 2019, with Pernambuco ranking sixth among states in YLDs in 2019.^31^ In the present study, higher rates of YLDs were observed during the pandemic period, alongside increases in G2D, corroborating the hypothesis that these two indicators reflect a substantial burden of disease.

The present study has some limitations. The use of secondary data from information systems may involve inconsistencies in data completeness and quality, as well as the potential for underreporting. In addition, because this is an ecological study, associations observed at the aggregate level cannot be directly extrapolated to individuals, although this design is useful for generating hypotheses to be tested in future research.

Overall, the findings of the present study suggest weaknesses in early case detection and timely management of leprosy in Pernambuco. Despite improvements in primary health care coverage in recent years, the data indicate that basic health units continue to face challenges in diagnosing and managing cases at the early stages of the disease, falling short of WHO recommendations.

## Final considerations

The findings of the present study indicate that the COVID-19 pandemic was associated with disruptions in the detection and follow-up of leprosy cases in Pernambuco, contributing to underreporting, treatment interruptions, and an increased proportion of cases presenting with advanced disability (G2D). The increase in YLDs further underlines the consequences of delayed diagnosis and reduced access to care during the pandemic, highlighting pre-existing vulnerabilities in the management of neglected diseases that were exacerbated by the health crisis.

These findings have direct implications for the organization of services within the Brazilian National Health System (SUS), underscoring the need to strengthen primary health care, resume active case-finding strategies, improve early diagnosis, and prevent disabilities. They also support the incorporation of burden-of-disease indicators, such as YLDs, into routine leprosy surveillance and reinforce the importance of studies evaluating the long-term effects of the pandemic to inform more equitable and effective public policies for chronic and neglected diseases.

## Authors’ contributions

Andrezza Tayonara Lins Melo: Conceptualization, methodology, data analysis, writing — original draft.

Pauliana Valéria Machado Galvão: Supervision, methodology, writing — review and editing.

Carolina da Cunha Correia: Methodology, writing—review and editing.

## Funding

We confirm that the study was financed in part by the Coordenação de Aperfeiçoamento de Pessoal de Nível Superior – Brasil (10.13039/501100002322CAPES) – Finance Code 001.

## Ethics statement

The study was conducted using secondary public-domain data. Ethical approval was obtained in accordance with institutional and national regulations, when applicable.

## Conflicts of interest

The authors declare no conflicts of interest.
